# What Drives Abdominal Obesity in Peru? A Multilevel Analysis Approach Using a Nationally Representative Survey

**DOI:** 10.3390/ijerph191610333

**Published:** 2022-08-19

**Authors:** Akram Hernández-Vásquez, Kamyla M. Olazo-Cardenas, Fabriccio J. Visconti-Lopez, Antonio Barrenechea-Pulache

**Affiliations:** 1 Centro de Excelencia en Investigaciones Económicas y Sociales en Salud, Vicerrectorado de Investigación, Universidad San Ignacio de Loyola, Lima 15024, Peru; 2 Facultad de Ciencias de la Salud, Universidad Científica del Sur, Lima 15067, Peru; 3 Department of Health Sciences, Universidad Peruana de Ciencias Aplicadas, Lima 15023, Peru

**Keywords:** Peru, abdominal obesity, multilevel analysis, epidemiology, health surveys

## Abstract

Abdominal obesity (AO) is a serious public health threat due to its increasing prevalence and effect on the development of various non-communicable diseases. A multilevel analysis of the 2019 Demographic and Family Health Survey (ENDES in Spanish) using the Latin American Diabetes Association (ALAD in Spanish) cut-off points was carried out to evaluate the individual and contextual factors associated with AO in Peru. A total of 30,585 individuals 18 years and older were included in the analysis. The prevalence of AO among Peruvians in 2019 was 56.5%. Individuals of older age (aOR 4.64; 95% CI: 3.95–5.45), women (aOR 2.74; 95% CI: 2.33–3.23), individuals with a higher wealth index (aOR 2.81; 95% CI: 2.40–3.30) and having only secondary education (aOR 1.45; 95% CI: 1.21–1.75) showed increased odds of presenting AO compared to their peers. At a contextual level, only the Human Development Index (aOR 1.59; 95% CI: 1.17–2.16) was associated with the development of AO. A high Human Development Index is the contextual factor most associated with AO. It is necessary to formulate and implement new public health policies focused on these associated factors in order to reduce the prevalence of OA and prevent the excessive burden of associated noncommunicable diseases.

## 1. Introduction

Abdominal obesity (AO), defined as the accumulation of fat in the abdominal region and measured through waist circumference/perimeter (measured in cm), is associated with several non-communicable diseases, such as cardiovascular diseases, diabetes, cancer, and chronic respiratory diseases [1]. This measure has been found to correlate better with arterial stiffness and major adverse cardiovascular events compared to Body Mass Index (measured in kg/m^2^) [2,3]. A meta-analysis reports that between 1985 and 2014 the prevalence of obesity worldwide in subjects aged 15 to 40 years increased from 16.3% to 33.9% [4]. In the United States and Portugal, AO reaches 47.2% and 50.5%, respectively [5,6], while in Mexico a prevalence of 81.6% was reported [7]. Using abdominal circumference as a measure for obesity, a prevalence of AO equal to 73.8% was estimated in Peru using the International Diabetes Federation criteria [8]. This prevalence is high compared to other countries, which is very alarming in the current context of the COVID-19 pandemic; because it has been shown that people with OA have a higher risk of developing a severe disease and a higher risk of mortality.

At the individual level, studies indicate that gender, age, level of physical activity and educational level are risk factors for developing AO. Due to a combination of cultural and hormonal factors, women have been reported to have a higher risk of developing AO compared to men [4,8,9,10]. AO is also associated with the age of the individual, with people over 40 years of age having a higher prevalence than young adults aged 15–40 years (48.0 vs. 23.8%) [4]. However, a systematic review and meta-analysis found that AO was twice as high in young adults compared to older subjects [4]. In addition, a sedentary lifestyle is considered a main factor associated with AO, with lower energy expenditure producing an increase in abdominal perimeter which does not alter BMI [11,12]. Similarly, AO is more prevalent in people with a higher educational level [13,14].

The characteristics of the environment in which the individual resides also have an impact on AO. For example, urban areas in low- and middle-income countries (LMIC), as well as capital cities, have reported a higher prevalence of AO [4]. One study reported that in households with greater food insecurity, there is a greater probability of developing central obesity in adolescents [15]. Additionally, environmental contaminants, such as dioxin, were found to be associated with an increased risk of AO in adults [16]. On the other hand, a study showed that places with a lower economic income and a higher unemployment rate were associated with a greater likelihood of developing AO [17]. Finally, several studies indicate that there is an inverse association between the level of greenness in residential zones and the probability of developing AO [18].

Since individual behaviors are also affected by the environment in which the individual develops, this can be studied using methods that allow capturing a hierarchical structure in the factors associated with the origin of AO. Low- and middle-income countries have shown to be at increased risk for AO development. However, in Latin America and the Caribbean (LAC) there are few studies on the influence of individual and contextual factors related to AO. In 2021, a study carried out in Peru found that socioeconomic characteristics have a great impact on the distribution of AO, with this alteration being concentrated in some geographical areas [8]. In this sense, the development of a multilevel model would allow examination of the impact of characteristics such as the Human Development Index (HDI), the natural region and the Food Vulnerability Index (FVI) on the development of AO in Peru. Currently, there are no validated cut-off points to define AO in Peru and some others LAC countries; however, the Latin American Diabetes Association (ALAD in Spanish) has recommendations that are used in Peru [19,20]. Therefore, this article aimed to analyze the individual and contextual factors associated with AO in Peru using the ALAD cut-off point.

## 2. Materials and Methods

### 2.1. Data and Sampling Design

This study was based on secondary analysis of the data collected in the 2019 Demographics and Health Survey (ENDES—acronym in Spanish), carried out by the National Institute of Statistics and Informatics of Peru (INEI). Comprehensive information on the methods used in this study has been described elsewhere [21]. Briefly, the 2019 ENDES employed a two-stage probabilistic, balanced and independent sampling, stratified at the department level and by rural-urban area, and produced a nationally representative sample. First, 1654 primary sampling areas (PSA), including 421 PSAs in the rural area and the remaining in the urban area, were selected with a probability proportional to size, in terms of its weight in occupied dwellings using a systematic random sampling. Second, using balanced sampling considering the variables of children under 5 years of age and women of childbearing age, a systematic sample of 18345 households were selected from the PSAs. We followed the “Strengthening the Reporting of Observational Studies in Epidemiology” (STROBE) statement for conducting this study and writing the manuscript [22].

### 2.2. Population

**Figure 1:**
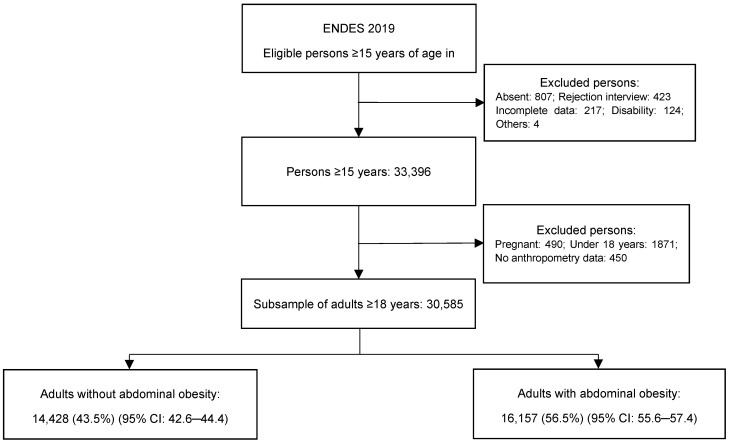
Flowchart of the selection of adults included in the study.

The study population was defined as adults aged 18 years or older, dwellers of urban and rural households selected by the sampling design. We included individuals who had complete information on the variables of interest in the present study. We excluded bedridden individuals, pregnant women, and individuals with physical disabilities that prevent measurement for the outcome of interest. Flowchart of the selection of adults included in the study is shown in Figure 1.

### 2.3. Data Collection

During the 2019 DHS survey, anthropometric measurements of individuals were collected. Waist circumference measurements were objectively collected by trained field technicians using standard techniques. Waist circumference was measured using standardized measuring tapes and boards with an accuracy of 0.1 cm.

### 2.4. Outcome of Interest

The outcome variable was AO (yes/no). We defined the presence of AO as a waist circumference ≥ 94 cm in men and ≥88 cm in women. The definition is based on the ALAD cut-off points [23]; measurements were made by trained anthropometrists using a 2-m-long metal measuring tape after exhalation and with the individual fasting for 2 h after eating. Further details of the measurement procedure can be found in the ENDES Anthropometrist Manual [24].

### 2.5. Individual-Household Level Characteristics

After reviewing relevant literature [8,25,26], the following variables were treated as individual-level characteristics: sex (women/men), age (18–29 years/30–59 years/60 years or more), educational level (no formal schooling/primary/secondary/high school), household wealth (poorest/poorer/middle/wealthier/wealthiest) and place of residence (urban/rural). To construct the household wealth index, the principal component analysis was used to assign each household a score depending on the ownership of selected durable assets and the characteristics of the dwelling [27].

### 2.6. Contextual-Level Characteristics

Contextual-level characteristics were reported in clusters (departments), except for the natural region that was taken as is. We included the 2019 HDI [28] and the 2018 FVI by departments, which collectively assessed the department’s wealth [29]. The HDI is a summary measure of average achievement in key dimensions of human development: education, health and income. A value closer to 1 indicates a higher level of human development within the territory [28]. The FVI is a measure that captures the food vulnerability of a given population; a value close to 1 is interpreted as greater vulnerability to food insecurity [29]. The index is constructed based on 24 distinct indicators that together give an overview of the availability, accessibility and utilization of food in a given department, or district within Peru [29].

### 2.7. Statistical Analysis

The data were analyzed with Stata version 17 (StataCorp., College Station, TX, USA). A spatial distribution map was prepared using the Quantum Geographic Information System (QGIS Desktop, version 3.24). The survey-specific procedures for weighting, clustering and stratification in the survey design were used in the analysis. Three basic steps were followed to analyze the data. The first step was the use of absolute and weighted frequencies to describe the sample. In the second step, we performed a bivariate analysis between the outcome of interest and the individual-level and contextual-level characteristics. We used the Rao-Scott chi-square to test differences in the outcome of interest between groups. Variables that were statistically significant in bivariate analyses at an α  =  0.05 were retained for a multilevel analysis.

In the last step, we ran a two-level multivariable logistic regression to assess the individual and contextual factors associated with AO. A two-tailed Wald test at a significance level of α = 0.05 was used to determine the statistical significance of the determinants. Natural regions were considered as a random effect to account for the unexplained variability at the contextual level. We fitted four models. Firstly, we fitted the Empty model or Null model that had no predictors (random intercept). This model was used to decompose the total variance of AO between the contextual and individual levels. Afterwards, Model 1 was fitted and contained only the individual-level variables. Then, Model 2 was fitted and included individual-level variables and an interaction between sex and age. Lastly, Model 3 was fitted and included both individual-level, interaction, and contextual-level characteristics. For all models, we presented the adjusted odds ratio (OR) and associated 95% confidence intervals (95% CI). These models were fitted by a Stata command “melogit” and allowed exploring the influence of contextual variables on the likelihood of AO. The equations of the fixed model (level 1) and random model (level 2) are described as follows:

(1) $$Y_{ij}=\beta_{0j}+\beta_{1j}X_{ij}$$

Equation (1) denotes a simple level 1 model with one individual-level predictor, where $Y_{ij}$ represents the log odds of AO for individual i in department j. $\beta_{0j}$ denotes the intercept or the average log odds of the occurrences of AO in department j. $X_{ij}$ is an individual level (level 1) predictor for individual i in department j. $\beta_{1j}$ is the slope associated with $X_{ij}$ showing the relationship between the individual-level variables and the log odds of AO occurrences [30].

The simple level 2 model with one individual level predictor is the following:

(2) $$\begin{array}{c} \beta_{0j}=\gamma 00+\gamma 01W_{j}+\varepsilon_{0j}\\ \beta_{1j}=\gamma 10\; \end{array}$$

In Equation (2), γ00 is the log odds of AO in department j. $W_{j}$ is a department-level predictor for department j, while γ01 is the slope associated with this predictor. The error term $\varepsilon_{0j}$ represents a unique effect associated with department j, and γ10 is the average-effect of the individual-level predictor [30]. Lastly, we estimated the following equation:

(3) $$Y_{ij}=\gamma 00+\gamma 10X_{ij}+\gamma 01W_{j}+\varepsilon_{0j}$$

Equation (3) combines Equations (1) and (2) by replacing the values of $\beta_{0j}$ and $\beta_{1j}$ as presented in Equation (2) into level 1. The probable contextual effects (random effects) were expressed in terms of intracluster correlation (ICC), median odds ratio (MOR) and percentage change in variance (PCV %) [30]. ICC represents the proportion of the variance at the group level divided by the sum of individual and group variance levels. We estimated the ICC using the linear threshold according to the formula used by Snijders and Bosker [31]. Mathematically, ICC was calculated as follows:

(4) $$\mathrm{ICC}=V_{A}/\left(V_{A}+V_{I}\right)$$

In Equation (4), $V_{A}$ refers to the variance area and $V_{I}$ denotes the individual-level variance. As the outcome of interest is not normally distributed, we corrected the ICC by estimating:

(5) $$\mathrm{ICC}=V_{A}/\left(V_{A}+3.29\right)$$

On the other hand, the MOR quantifies the variation between clusters by comparing two persons from two different randomly chosen clusters. The purpose of the MOR is to translate the variance area in the widely used OR scale, which has a steady and intuitive interpretation. The MOR is defined as the median value of the OR between the area at highest risk and the area at lowest risk. When randomly picking out two areas, the MOR can be conceptualized as the increased risk (in median) if moving to another area with a higher risk [30]. The MOR was calculated using the following formula:

(6) $$\mathrm{MOR}=exp\left[\sqrt{\left(2XV_{A}\right)\times 0.6745}\right]≃exp\left(0.95\sqrt{V_{A}}\right)$$

In Equation (6), 0.6745 denotes the 75th centile of the cumulative distribution function of the normal distribution with mean 0 and variance 1 [32]. Further details to estimate the MOR and PCV are described elsewhere [32].

The PCV% was calculated using the following formula:

(7) $$\mathrm{PCV}=\left(V_{A}-V_{B}\right)/V_{A}\times 100$$

In Equation (7), $V_{A}$ is the variance of the initial model (empty model) while $V_{B}$ represents the variance of the subsequent model (model with more terms). Further details of PCV can be found elsewhere [33].

For model comparison, we used the log-likelihood ratio and the Akaike Information Criteria (AIC) test. The highest log-likelihood and the lowest AIC indicate the best-fit model. Multicollinearity was tested using the variance inflation factor (VIF). All VIF values were reported as being <10, thus no multicollinearity constraints were detected in the regression models.

### 2.8. Ethical Considerations

Informed consent was obtained at the beginning of each interview and the database is freely available at the website of the INEI. This study did not require the approval of an ethics committee because it was an analysis of secondary data that is in the public domain and does not allow the evaluator participants to be identified.

## 3. Results

The characteristics of the study participants are shown in Table 1. A total of 30,585 individuals were included in the analysis. Nearly half of the participants were women (51.2%), and 54.8% of the overall participants were between 30 and 59 years old. Secondary school was completed by 40% of the study participants, and most of the individuals lived in an urban setting (81%). According to the contextual variables, most of the individuals lived on the coast (63%) and nearly half of the overall participants lived in departments with a low FVI (51.7%).

**Figure 2:**
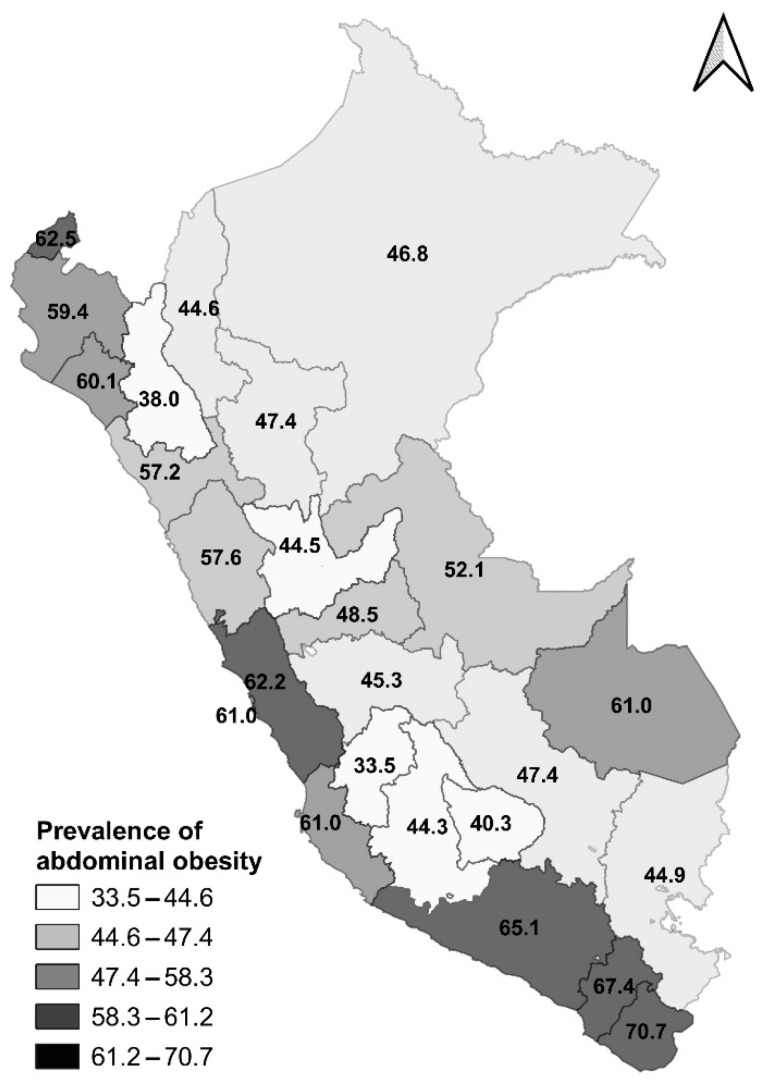
Prevalence of abdominal obesity according to the departments of Peru.

The prevalence of AO according to the departments of Peru is shown in Figure 2 and Table S1. The highest prevalence of AO was found in Tacna (70%), followed by Moquegua (70%), Arequipa (70%) and Tumbes (60%). On the other hand, Huancavelica (30%), Cajamarca (40%) and Apurimac (40%) reported the lowest prevalence of AO.

Adjusted fixed effects of individuals -and department-level factors on the risk of AO are shown in Table 2 and model 3. At the individual level, women showed increased odds (adjusted OR [aOR] 2.74 [95% CI 2.33–3.23]) of having AO compared to men. The odds of having AO also increased in individuals aged 30-59 years old (aOR 4.35 [95% CI 3.95–4.79]) and in individuals aged 60 years or more (aOR 4.64 [95% CI 4.35–5.49]) compared to those 29 years old or younger. Participants with a primary (aOR 1.43 [95% CI 1.23–1.67]) or secondary (aOR 1.45 [95% CI 1.21–1.75]) education had higher odds of having AO compared to those with no formal schooling. We found a positive relationship between AO and the wealth index. Furthermore, individuals from rural (aOR 0.85 [95% CI 0.77–0.94]) settings showed reduced odds of having AO compared with those from urban settings. At the community level, the odds of having AO were increased when the HDI increased.

## 4. Discussion

We set out to analyze the individual and contextual factors associated with AO in Peru using the ALAD cutoff points. We found that the prevalence of AO among Peruvians in 2019 was 56.5%. Individuals of older age, women, those with the highest wealth index and having only a secondary education had increased odds of presenting AO compared to their peers. Furthermore, higher department HDI was associated with the development of AO.

Similar to the findings of Farro-Maldonado and colleagues, the main individual factors that influence AO among Peruvians are age, female sex, wealth index and having a secondary education [8]. These associations could be due to a combination of physiological, economic and sociocultural factors. Older individuals have decreased daily energy expenditure and decreased free-fat mass, especially if they acquire other chronic diseases that limit mobility, leading to a corresponding increase in abdominal perimeter and AO [35,36]. Industrialization and urbanization have likely led more educated individuals in developing countries to adopt more sedentary lifestyles. These lifestyles involve prolonged hours in front of a screen, increased exposure to fast food and an increased availability of motorized vehicles with limited spaces for individuals to engage in physical activity in the open air [37,38]. Peruvian women may be especially vulnerable to AO as they may gain more weight after pregnancy, and have less incentive to pursue a professional career due to the lower wages and traditional gender norms still prevalent in rural areas of the country [39]. Thus, diverse individual factors likely influence the development of AO throughout Peru. 

The HDI was the contextual factor with the highest association of AO. Increases in the gross domestic product (GDP) per capita in LMIC predict an increase in the BMI, owing largely to the widespread nutritional transitions that occur with development [40]. In Peru, the urbanization of rural life has caused a shift from traditional diets that were mostly self-grown to high-energy market-bought foods with larger portions and lower nutritional value [41,42]. In 2020, on average, inhabitants of rural environments consumed fruits and vegetables less frequently than in urban settings (4.1 vs. 4.6 days of the week) [43]. Fast food advertisements on social media could also hinder the ability of young adults to opt for healthy eating behaviors [44]. Workers in rotating shifts, temporary contacts, and manual labor consume fewer fruits and vegetables, which is associated with longer working days, lower educational attainment, and higher levels of stress [45]. Since around 18.9% of the Peruvian GDP is produced by the informal sector (composed of >70% of the economically active population) [46], a large sector of the working force is at increased risk of AO. Thus, urbanization has likely led to changes in food availability and eating behaviors of a vastly informal population, leading to increasing levels of AO. 

Despite the coastal region having the highest prevalence of AO (61.4%), we did not find a significant association between obesity and natural region. Its effect may be obscured by the overall high prevalence of AO (≥50%) across most departments and the differences in the prevalence of AO related to area of residence. Each department has varying levels of economic growth, with the most development occurring in the capital, Lima, and other coastal departments such as Arequipa and La Libertad which produced 43.5%, 5.4% and 4.4% of the national GDP in 2020 [47]. Departments with the highest HDI (>0.61) are in the coast, while those with the five lowest HDI (<0.45) are located predominantly in the highlands. These developed areas are more globalized, and its residents live in an obesogenic environment [28]. Despite the improvements of the economy and health indicators, inequality in access to health care persists [48]. In 2020, 97% of the 8783 primary care centers throughout the nation had inadequate infrastructure or resources to meet the needs of the population [49]. Over half of these centers do not have a medical doctor; and many are concentrated in coastal cities and in areas with higher economic development [50]. Peri-urban and rural settings are especially susceptible to both malnutrition and obesity [41]. Thus, rural inhabitants of the highlands and jungle have limited access to preventive health care services, and therefore, do not receive education regarding the health risks of excessive body fat and the increased risk of developing additional chronic diseases.

We found that Peruvians with low FVI have highest prevalence of obesity. A prior systematic review found that the relationship between food insecurity and weight abnormalities is modified by the socioeconomic level of the country. Individuals from low-income countries and high food insecurity had significant risk of underweight, while the opposite occurs in high income countries [51]. In Peru food insecurity is related primarily to poverty and living in a rural area, which has lower access to marketplaces, education, higher paying jobs and basic services such as drinkable water [29]. In 2020 only 13,8% of households in rural areas had access to a refrigerator and around 24.1% still didn’t have access to safe water in their household [52]. Overall, in Peru, city development reduces food insecurity, thus FVI has been found to be lower in coastal areas compared to the jungle and highlands; this association persists even at the provincial and district level. “Urban poverty” may also contribute to obesity in low FVI areas. Poor individuals in urban settings depend more on supply and access chains, are more exposed to ultra-processed foods, have weaker networks to allow sharing of food and need to allocate a more resources to food while being vulnerable to any external shock. The difficulties these persons face can also lead to mental health problems that contribute to weight change through changes in release of hormones and neurotransmitters [53]. Thus, socioeconomic disparities in Peru are likely predisposing poor individuals in low FVI areas to become obese, while those in high FVI areas face malnutrition and underweight. 

Among the limitations of our study, we must highlight that we were unable to establish causal relationships due to the use of cross-sectional data. As this study is a secondary analysis of a survey, we can only include the existing variables in our analysis. We had no access to weighted proportions for area of residence, thereby limiting the stratification of our analysis by this variable and potentially affecting the associations found. We were also unable to evaluate additional factors of interest, such as women’s empowerment or regional access to electronic media. Despite these limitations, this study employs a nationally representative data set that it is the only source of information currently available in Peru on AO.

In conclusion, approximately 6 out of 10 Peruvians presented OA. Individual factors associated with this are sex, age, wealth index and education level. At the contextual level, the HDI was associated with AO. Developing cities that are transitioning from low HDI should be monitored by policy makers to avoid the creation of obesogenic environments and loss of healthy habits. While coastal cities which already have high prevalence of AO should promote more active lifestyles in the big city through new public health policies. These could target women, those aged 30 years and above and areas that concentrate inhabitants with lower educational attainment. These interventions would reduce the prevalence of OA, preventing the excessive burden of associated noncommunicable diseases.

## Figures and Tables

**Table 1:** Sample characteristics.

Characteristics	n (%)	Abdominal Obesity		*p*-Value
		No	Yes	
		% (95% CI)	% (95% CI)	
**Overall**	30,585 (100)	43.5 (42.6–44.4)	56.5 (55.6–57.4)	
**Individuals**				
Sex				
Man	13,199 (48.8)	51.9 (50.5–53.2)	48.1 (46.8–49.5)	<0.001
Woman	17,386 (51.2)	35.5 (34.4–36.7)	64.5 (63.3–65.6)	
Age				
18–29 years	8436 (26.8)	67.5 (65.8–69.1)	32.5 (30.9–34.2)	<0.001
30–59 years	17,096 (54.8)	34.8 (33.7–36.0)	65.2 (64.0–66.3)	
60 or more	5053 (18.3)	34.3 (32.3–36.4)	65.7 (63.6–67.7)	
Education level				
No formal schooling	1624 (3.9)	45.8 (42.1–49.7)	54.2 (50.3–57.9)	0.021
Primary	7762 (20.6)	41.5 (39.8–43.3)	58.5 (56.7–60.2)	
Secondary	12,048 (39.6)	44.9 (43.4–46.3)	55.1 (53.7–56.6)	
Higher	9151 (35.9)	42.8 (41.2–44.5)	57.2 (55.5–58.8)	
Wealth Index				
Poorest	9797 (18.3)	62.1 (60.6–63.6)	37.9 (36.4–39.4)	<0.001
Poor	7787 (21.2)	48.0 (46.3–49.7)	52.0 (50.3–53.7)	
Medium	5530 (20.6)	40.8 (38.9–42.7)	59.2 (57.3–61.1)	
Rich	4222 (19.9)	34.4 (32.3–36.5)	65.6 (63.5–67.7)	
Richest	3249 (20.0)	33.5 (31.3–35.9)	66.5 (64.1–68.7)	
Area of residence				
Urban	19,822 (80.9)	39.9 (38.8–40.9)	60.1 (59.1–61.2)	<0.001
Rural	10,763 (19.1)	58.8 (57.2–60.3)	41.2 (39.7–42.8)	
**Contextual Factors**				
Department HDI				
Low	10,670 (17.6)	57.4 (56.0–58.7)	42.6 (41.3–44.0)	<0.001
Medium	9936 (32.2)	45.4 (44.1–46.7)	54.6 (53.3–55.9)	
High	9979 (50.3)	37.4 (35.9–38.9)	62.6 (61.1–64.1)	
Natural Region				
Jungle	5636 (8.3)	51.8 (50.1–53.5)	48.2 (46.5–49.9)	<0.001
Highlands	12,629 (28.8)	51.8 (50.5–53.0)	48.2 (47.0–49.5)	
Coast	12,320 (62.9)	38.6 (37.3–39.9)	61.4 (60.1–62.7)	
Food vulnerability index				
Low	11,091 (51.7)	37.7 (36.3–39.2)	62.3 (60.8–63.7)	<0.001
Medium	10,127 (27.1)	47.4 (45.9–48.9)	52.6 (51.1–54.1)	
High	9367 (21.3)	52.6 (51.3–53.9)	47.4 (46.1–48.7)	

Estimates include the weights and ENDES 2019 sample specifications. The *p*-value was calculated using the Rao-Scott Chi-squared. 95% CI: 95% confidence interval. HDI: Human Development Index.

**Table 2:** Multilevel modeling results.

	Empty Model		Model 1		Model 2		Model 3	
Variables	OR	*p* Value	OR (95% CI)	*p* Value	OR (95% CI)	*p* Value	OR	*p* Value
**Individual-level variables**								
Sex								
Man			Reference		Reference		Reference	
Woman			2.48 (2.14–2.87)	<0.001	2.74 (2.33–3.23)	<0.001	2.74 (2.33–3.23)	<0.001
Age								
18–29 years			Reference		Reference		Reference	
30–59 years			4.13 (3.91–4.36)	<0.001	4.34 (3.94–4.79)	<0.001	4.35 (3.95–4.79)	<0.001
60 o more			4.01 (3.51–4.58)	<0.001	4.64 (3.95–5.46)	<0.001	4.64 (3.95–5.45)	<0.001
Education Level								
No formal schooling			Reference		Reference		Reference	
Primary			1.52 (1.28–1.80)	<0.001	1.44 (1.24–1.68)	<0.001	1.43 (1.23–1.67)	<0.001
Secondary			1.55 (1.26–1.91)	<0.001	1.47 (1.22–1.77)	<0.001	1.45 (1.21–1.75)	<0.001
Higher			1.28 (1.01–1.61)	0.031	1.20 (0.97–1.49)	0.068	1.20 (0.97–1.48)	0.065
Wealth Index								
Poorest			Reference		Reference		Reference	
Poor			1.83 (1.66–2.02)	<0.001	1.83 (1.66–2.02)	<0.001	1.82 (1.65–2.00)	<0.001
Medium			2.31 (2.07–2.58)	<0.001	2.33 (2.09–2.59)	<0.001	2.28 (2.05–2.53)	<0.001
Rich			2.87 (2.44–3.38)	<0.001	2.89 (2.46–3.41)	<0.001	2.81 (2.40–3.30)	<0.001
Richest			2.74 (2.30–3.27)	<0.001	2.76 (2.32–3.30)	<0.001	2.65 (2.22–3.17)	<0.001
Area of residence								
Urban			Reference		Reference		Reference	
Rural			0.83 (0.76–0.92)		0.83 (0.76–0.92)		0.85 (0.77–0.94)	
Sex#Age								
Woman # 18–29 years					Reference		Reference	
Woman # 30–59 years					0.92 (0.80–1.06)	0.232	0.92 (0.80–1.06)	0.236
Woman # 60 o more					0.73 (0.63–0.85)	<0.001	0.73 (0.63–0.85)	<0.001
**Contextual Factors**								
Department HDI								
Low							Reference	
Medium							1.22 (0.99–1.49)	0.056
High							1.59 (1.17–2.16)	0.003
Natural Region								
Jungle							Reference	
Highlands							0.81 (0.69–0.95)	0.011
Coast							0.98 (0.79–1.21)	0.852
Food Vulnerability Index								
Low							Reference	
Medium							1.13 (0.93–1.38)	0.214
High							1.03 (0.87–1.22)	0.751
N	30,585		30,585		30,585		30,585	
Community-level variance (SE)	0.15 (0.03)		0.06 (0.02)		0.06 (0.02)		0.01 (0.01)	
Fitness model statistics (AIC)	16,652.88		14,825.01		14,822.55		14,805.4	
ICC (%)	0.0444855		0.0188607		0.018835		0.0046789	
Log-likelihood	−8324.44		−7399.5073		−7396.276		−7382.7019	
LR Test								
PCV (%)			0.59		0.00		0.76	
Median odds ratio	1.45		1.27		1.27		1.13	
−2 log likelihood	16,648.879		14,807.12		14,792.553		14,765.404	

The proportional change in variance (PCV) expresses the change in the area level variance between the empty model and the individual level model, and between the individual level model and the model further including the area level covariate [33]. Estimates include the weights by method A for scaling the weights recommended by Carle [34]. Empty model had no predictors (random intercept). Model 1 was fitted and contained only the individual-level variables. Model 2 was fitted and included individual-level variables and an interaction between sex and age. Model 3 was fitted and included both individual-level, interaction, and contextual-level characteristics. HDI: Human Development Index; OR: odds ratio; 95% CI: 95% confidence interval; ICC: intracluster coefficient; SE: standard error; LR: likelihood ratio; PCV: percentage change in variance; AIC: Akaike Information Criteria; #: interaction.

## Data Availability

The data from the ENDES are publicly accessible on the INEI website: http://iinei.inei.gob.pe/microdatos/ (accessed on 13 June 2022).

## Supplementary Materials

The following supporting information can be downloaded at: https://www.mdpi.com/article/10.3390/ijerph191610333/s1. Table S1. Prevalence of abdominal obesity according to the departments of Peru.
