# Supplementary material for: What Drives Abdominal Obesity in Peru? A Multilevel Analysis Approach Using a Nationally Representative Survey
Source: Int J Environ Res Public Health. 2022 Aug 19;19(16):10333. doi: 10.3390/ijerph191610333 (PMC9407803; doi:10.3390/ijerph191610333)
Supplement: Supplementary file 1 [file ijerph-19-10333-s001.zip › ijerph-1793465-supplementary.pdf]

**Table S1.** Prevalence of abdominal obesity according to the departments of Peru.

| Departments | Prevalence | 95% CI |
|---|---|---|
| Amazonas | 44.6 | 41.3 – 48.0 |
| Ancash | 57.6 | 54.1 – 61.2 |
| Apurimac | 40.3 | 36.6 – 44.0 |
| Arequipa | 65.1 | 61.9 – 68.2 |
| Ayacucho | 44.3 | 40.9 – 47.8 |
| Cajamarca | 38.0 | 35.3 – 40.6 |
| Callao | 61.0 | 57.9 – 64.1 |
| Cusco | 47.4 | 43.0 – 51.9 |
| Huancavelica | 33.5 | 30.4 – 36.7 |
| Huanuco | 44.5 | 40.8 – 48.2 |
| Ica | 61.0 | 57.5 – 64.5 |
| Junin | 45.3 | 41.3 – 49.2 |
| La Libertad | 57.2 | 53.4 – 61.0 |
| Lambayeque | 60.1 | 57.2 – 63.0 |
| Lima | 62.2 | 60.3 – 64.2 |
| Loreto | 46.8 | 43.8 – 49.7 |
| Madre de Dios | 61.0 | 57.1 – 64.9 |
| Moquegua | 67.4 | 64.4 – 70.5 |
| Pasco | 48.5 | 44.9 – 52.0 |
| Piura | 59.4 | 56.2 – 62.5 |
| Puno | 44.9 | 40.2 – 49.6 |
| San Martin | 47.4 | 43.8 – 50.9 |
| Tacna | 70.7 | 67.6 – 73.8 |
| Tumbes | 62.5 | 59.4 – 65.6 |
| Ucayali | 52.1 | 48.8 – 55.5 |
